# Mitochondrial Dysfunction and Disturbed Coherence: Gate to Cancer

**DOI:** 10.3390/ph8040675

**Published:** 2015-09-30

**Authors:** Jiří Pokorný, Jan Pokorný, Alberto Foletti, Jitka Kobilková, Jan Vrba, Jan Vrba

**Affiliations:** 1 Institute of Photonics and Electronics, Czech Academy of Sciences, Chaberská 57, 182 51 Prague 8, Czech Republic; 2 Institute of Physics, Czech Academy of Sciences, Na Slovance 2, 182 21 Prague 8, Czech Republic; E-Mail: pokorny@fzu.cz; 3 Institute of Translational Pharmacology, National Research Council—CNR, Via Fosso del Cavaliere 100, Rome 00133, Italy; E-Mail: contact@albertofoletti.ch; 4 Clinical Biophysics International Research Group, via Maggio 21, Lugano 6900, Switzerland; 5 Department of Obstetrics and Gynaecology, 1st Faculty of Medicine, Charles University in Prague, Apolinářská 18, 128 00 Prague 2, Czech Republic; E-Mail: jitka.kobilkova@centrum.cz; 6 Faculty of Electrical Engineering, Czech Technical University in Prague, Technická 2, 166 27 Prague 6, Czech Republic; E-Mail: vrba@fel.cvut.cz; 7 Faculty of Biomedical Engineering, Czech Technical University in Kladno, Sitná Square 3105, 272 01 Kladno, Czech Republic; E-Mail: jan.vrba@fbmi.cvut.cz

**Keywords:** cancer biophysics, mitochondrial dysfunction, water ordering, microtubule oscillations, disturbed coherence, LDH virus

## Abstract

Continuous energy supply, a necessary condition for life, excites a state far from thermodynamic equilibrium, in particular coherent electric polar vibrations depending on water ordering in the cell. Disturbances in oxidative metabolism and coherence are a central issue in cancer development. Oxidative metabolism may be impaired by decreased pyruvate transfer to the mitochondrial matrix, either by parasitic consumption and/or mitochondrial dysfunction. This can in turn lead to disturbance in water molecules’ ordering, diminished power, and coherence of the electromagnetic field. In tumors with the Warburg (reverse Warburg) effect, mitochondrial dysfunction affects cancer cells (fibroblasts associated with cancer cells), and the electromagnetic field generated by microtubules in cancer cells has low power (high power due to transport of energy-rich metabolites from fibroblasts), disturbed coherence, and a shifted frequency spectrum according to changed power. Therapeutic strategies restoring mitochondrial function may trigger apoptosis in treated cells; yet, before this step is performed, induction (inhibition) of pyruvate dehydrogenase kinases (phosphatases) may restore the cancer state. In tumor tissues with the reverse Warburg effect, Caveolin-1 levels should be restored and the transport of energy-rich metabolites interrupted to cancer cells. In both cancer phenotypes, achieving permanently reversed mitochondrial dysfunction with metabolic-modulating drugs may be an effective, specific anti-cancer strategy.

## 1. Introduction

A continuous energy supply to biological systems along with its transformation are among the essential processes for life. Energy is required for membrane and organelle assembly, ion separation, formation of electric potential differences, excitation of vibrations, *etc*. Energy supply enables formation and maintenance of structures and states far from the thermodynamic equilibrium. Energy stored in oscillating systems represents a source of forces for biological use. The organization of bodies with macroscopic dimensions and the synchronization of mutually dependent processes require forces of corresponding range. H. Fröhlich formulated a hypothesis of coherent electric polar vibrations in biological systems with energy condensation in a mode of motion and correlated over macroscopic regions [1,2,3,4,5]. The disturbance of cell interactions within a tissue due to frequency changes in the electric polar vibrations was assumed to be an initial condition for cancer local invasion and metastasis [6]. Electric polarity is a specific feature of the majority of biological molecules, which are electric dipoles and/or multipoles, and vibrations generate an electromagnetic (EMG) field. The organization units of eukaryotic cells (*i.e.*, microtubules) are electric polar structures with energy supply.

It has been postulated that any vibration is highly damped by water, due to its high content within the cell (approximately 70%). The theoretical analysis and experimental investigation of exclusion zones at the hydrophilic surfaces have proven the ordering of water molecules, which depends on special properties of water molecules in a range of physiological temperatures. Water forms coherent domains (CD) whose linear dimension is about 80–100 nm: in a strong electric field, the CDs are aligned and a layer of ordered CDs is formed. The layers of ordered CDs (*i.e.*, layers of ordered water molecules) are formed around structures with hydrophilic surfaces including microtubules and mitochondria, and may have macroscopic dimensions and possess elastic properties similar to a gel.

As energy supply is a necessary condition for life, and any disturbance of energy metabolism likely leads to a pathological state. O. Warburg provided evidence for the defective processes of energy transformation in cancer. Experimental evidence he provided demonstrated that cancer cells can obtain approximately the same amount of energy from fermentation as from respiration, whereas normal cells obtain greater levels of energy from oxidation than from fermentation [7,8]. He also proved that in cancer, the impairment of oxidative metabolism, which displays disturbance of pyruvate transfer, is conditioned by mitochondrial dysfunction, and intuitively described the consequences as a structure type defect which is now explained as disturbance of water molecules’ ordering. He wrote that “…*it is immaterial to the cells whether they obtain their energy from respiration or from fermentation*…” and that “*The adenosine triphosphate synthesized by respiration therefore involves more structure than adenosine triphosphate synthesized by fermentation*.” Researchers in the past century focused predominantly on biochemical and genetic mechanisms, erroneously considering the Warburg effect as a side corollary of the cancer transformation rather than its central phenomenon.

Energy transformation in mitochondria produces a special state to condensate energy in electric vibrations of microtubules. The inner membrane potential is connected with forming layers of ordered water molecules around mitochondria, which provides low damping of vibrations and, therefore, enables their high excitation. The mitochondrial dysfunction reported by Warburg is caused by inhibition of pyruvate transfer into mitochondrial matrix. The number of protons transferred from the matrix is reduced, and in turn decreases the membrane potential and changes water ordering around mitochondria. The decrease of membrane potential results in increased damping and low condensed energy of vibrations.

The consequences of the Warburg effect have not been fully proved yet. The microtubule electric polar vibrations in cancer cells presenting the Warburg effect, or in fibroblasts presenting the reverse Warburg effect, may be highly damped. In nonlinear oscillation systems, the frequency depends on power. If cell interactions are mediated by the near EMG field, then changes of frequency disturb the attractive interactions and cells can leave their position. An extremely high electric field can also affect chemical reactions [9,10]. Yet, the effect of the coherent electric oscillations on cellular processes remains poorly understood.

## 2. Ordered Water

Water is the main component of living systems, and its extraordinary properties are undoubtedly necessary for the living state. The most interesting feature seems to be the mechanism exploiting the coherent dynamic interactions of water molecules with quantized, transverse EMG field [11,12,13]. A short overview of the theoretical analysis based on the quantum electrodynamics is presented.

Below a critical temperature, water molecules experience phase transition from interaction through collisions to a coherent oscillation state in CDs. Electron clouds of single molecules fluctuate in phase between two configurations (energy states) and interact with the EMG field within CDs whose dimensions correspond to the wavelength of the EMG field. The oscillation frequency in the CD is lower than the frequency of EMG oscillations of the same wavelength in vacuum, which prevents EMG losses by radiation outside the CD. The energy of a molecule in the coherent state is lower than in the incoherent state. The difference is known as “energy gap”. Coherent state is thus produced through a loss of energy. Supply of energy greater than the gap between the incoherent–coherent states can destroy the CD. The coherent oscillation performed by each molecule takes place between the fundamental state where the electrons are strongly bound (energy 12.60 eV for electron release and to ionize the molecule) and the excited state (with excitation energy of 12.06 eV). In the coherent state of water, a small amount of energy such as 0.54 eV can release an electron. Therefore, the coherent state is connected with a high tendency to yield free electrons. The incoherent state is liable to capture electrons and produces H_2_O^−^ ions. This effect is useful for redox reactions. Under the static electric field, for instance, at the hydrophilic surfaces, CDs are ordered in a layer and water properties are changed.

A biologically significant feature of a CD is its ability to collect “low-grade” energy with high entropy and transform it into “high-grade” energy with low entropy, by exciting coherent vortices of almost free electrons. CDs have a long life as the EMG radiation is prevented and energy losses most likely originate in a chemical way when the energy of coherent excitation is transferred to non-aqueous molecules causing chemical activation. At a given value of physiological temperatures, a fraction of water molecules is in a coherent state and the rest in an incoherent state.

**Figure 1:**
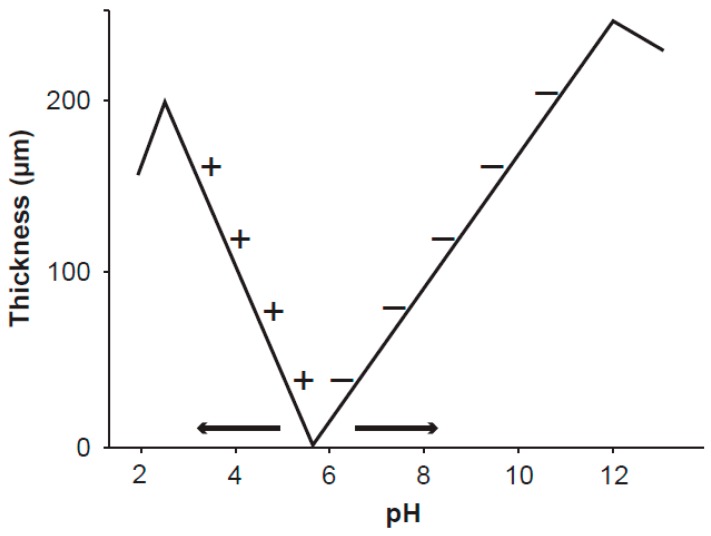
Schematic visualization of the thickness of the exclusion zone *versus* pH factor at the surface of polyvinyl alcohol gel [14]. The gel was stored in pure water at pH 5.7. The signs + and − denote positively and negatively charged particles suspended in aqueous solution at different pH and excluded from the exclusion zone. The exclusion zone is formed by the static electric field emanating from the gel surface charge. The measured curves are important for understanding the ordered water layers around mitochondria. As the inner membrane potential of mitochondria depends on the level of oxidative metabolism (*i.e.*, on the intensity of the static electric field), the working point shifts along the curve and very likely even the point of zero thickness may move as shown by the arrows. The shifts of the working point along the pH axis seem to explain Warburg’s “more structure” in normal cells.

At hydrophilic surfaces, water forms layers whose properties corroborate the theoretical findings for CDs. The first experimental evidence proving a 5–20 nm-thick empty layers around microtubules was reported by Amos [15]. The layer was called clear zone, as the solutes were expelled beyond its outer rim and its formation depends on the microtubule surface charge and opposite charges in water [16]. Layers of water molecules with no solutes were measured on hydrophilic surfaces and thereby they were called exclusion zones. These zones are macroscopic layers and their thickness may reach about 500 μm [17]. Physical properties of ordered water in the exclusion zones differ from the bulk water, which is a mixture of incoherent and coherent parts. In comparison with bulk water, the layers of ordered water exhibit higher viscosity [18], decreased thermal motion of molecules [17], and different pH [19]. Thickness of the exclusion zone depends on the pH of water [14] and most likely also on the charge of the hydrophilic surface, i.e., the intensity of the static electric field. Over a hydrophilic surface of polyvinyl alcohol gel and at a given value of pH of water (about 5.7), the exclusion zone is not formed. The thickness of the exclusion zone measured by the exclusion of positively (negatively) charged particles increases with decreasing (increasing) pH from the given value of pH (Figure 1). This phenomenon may explain differences between the apparent membrane potential measured by the uptake and retention of positively charged fluorescent dyes by mitochondria, and the true potential corresponding to mitochondrial oxidative metabolism.

Macroscopic water ordering can also be achieved by an electric field of external source. For instance, a floating water bridge between two glass beakers was set up after applying a strong electric field with the intensity of about 600–700 kV/m [20,21,22,23]. The length of the floating water bridge was 3 cm.

## 3. Mitochondria Condition Coherence

Mitochondria have multiple functions in living cells. They are of different shapes, with linear dimension of about 0.5–1 μm, and form approximately 22% of cellular cytoplasmic volume and mass. Their inner membrane, folded in numerous cristae, plays a fundamental role in mitochondrial activity. Their basic function is connected with oxidative metabolism, producing adenosine and guanosine triphosphate (ATP and GTP, respectively) for biological needs. Mitochondria also participate in the controlling of the apoptotic signaling cascade by which cells are directed to programmed death. However, besides energy production and apoptosis modulation, mitochondria perform another crucial task: water ordering.

Chemical energy is used for proton transfer from the matrix to the intermembrane space. Protons diffuse into the cytosol through porin channels in the outer membrane and form a charged layer around mitochondria. These processes are part of an important intermediate mechanism in energy production, but they are also significant for the creation of the coherent state of EMG oscillations. A layer of a strong static electric field created around mitochondria was measured up to a distance of about 2 μm [24]. At the mitochondrial membrane, the field intensity was about 3.5 MV/m, and displayed virtually linear dependence on the distance from the mitochondrial surface, which did not correspond to the distribution of protons in the potential layer [25]. Therefore, it may be reasonable to assume that water molecules in the cytosol around mitochondria are ordered in a similar way as if they were located around charged surfaces. The pH value is decreased by about 1 pH unit due to proton transfer. The membrane potential of a respiring mitochondria is about 140 mV and the proton motive force across the inner membrane is about 200 mV (a pH gradient is equivalent to a membrane potential of about 60 mV) [26]. As a result of water ordering, a dynamic phase transition occurs from a viscous liquid phase to an almost elastic gel which affects inner cellular processes.

A quantitative assessment of the thickness of the ordered water molecules layer around mitochondria enables determining its effect on the excitation of the coherent EMG field in living cells. For spherical mitochondria and spherical cells of about 0.5 μm and 10 μm in diameter, respectively, the average distance between 1000 uniformly distributed mitochondria is about 1 μm. Findings of Tyner *et al.* [24] proved that a strong electric field is formed around mitochondria at a distance greater than about 1 μm. Exclusion zones around mitochondria may mutually cross one over another. If fully functional mitochondria are aligned along microtubules, then water in this region is ordered, resulting in high excitation of microtubule oscillations. Disturbed water ordering around dysfunctional mitochondria may cause high damping, which correlates with Warburg’s pioneering suggestion [8].

**Figure 2:**
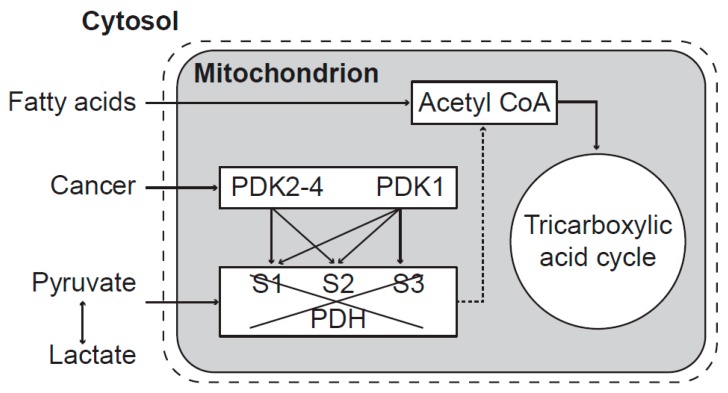
Mitochondrial dysfunction caused by inhibition of pyruvate transfer to mitochondria. The activity of the PDH complex is blocked by PDK enzymes. PDK1 can phosphorylate all three sites—S1, S2, and S3 (Ser-264, Ser-271, and Ser-203, respectively) whereas PDK2, PDK3, and PDK4 each phosphorylate only the sites S1 and S2. After phosphorylation, mitochondrial energy production depends only on fatty acids. Both isoenzymes of pyruvate dehydrogenase phosphatase (PDP1 and PDP2) can reactivate phosphorylated PDH enzymes [27]. Mitochondrial dysfunction transforms chemical changes into biophysical pathological processes along the transformation pathway to cancer.

Mitochondrial dysfunction is caused by inhibition of the pyruvate transfer into mitochondrial matrix through phosphorylation of pyruvate dehydrogenase enzymes (Figure 2).

## 4. Microtubules as Generators of EMG Field

In eukaryotic cells, microtubules form a filamentous structure which is the primary organizer of cytoskeleton. Microtubules are hollow tubes of a circular cross section, with the inner and outer diameter of 17 and 25 nm, respectively, described by Amos and Klug [28]. During the interphase, they grow outward from the centrosome, a spherical structure in the center of the cell, and form radial fibers. Some of them are attached to membrane structures. In the M phase, the microtubules of the mitotic spindle emanate from two centrosomes.

Microtubule physical characteristics comply with the requirements for generating the EMG field: they are electrically polar, nonlinear, and excited by energy supply. Tubulin heterodimers forming organized structure of microtubules are electric dipoles whose dipole moments are about 1000 Debye, *i.e.*, 10^−26^ cm [29,30]. Several mechanisms have been described for energy supply required to excite polar vibrations. Energy can be supplied by the hydrolysis of GTP to GDP upon β-tubulin polymerization [31,32], by energy losses through the motion of motor proteins along microtubules [33], and most likely also by non-utilized energy freed from mitochondria [34,35]. Photons released from chemical reactions may supply energy in the UV and visible wavelength region.

The Fröhlich’s hypothesis [1,2,3,4,5,6] is strongly supported by experimental results on mechanical vibrations in living cells. Pelling *et al.* [33,36] measured vibrations of yeast cells *Saccharomyces cerevisiae* at acoustical frequencies (1.63 and 0.87 kHz at temperatures of 30 and 22 °C, respectively). Dead cells did not exhibit any vibrational activity. Mechanical vibrations of the cell membrane of yeast cells and their electric activity in the acoustic frequency band were measured by Jelínek *et al.* [37]. The frequencies of mechanical vibrations coincided with the frequencies of electrical oscillations. Nanoscale vibrations are indicators of metabolic activity and a signature of life [38]. The majority of biological macromolecules and structures are electrically polar and, therefore, EMG activity also belongs to the signature of life.

**Figure 3:**
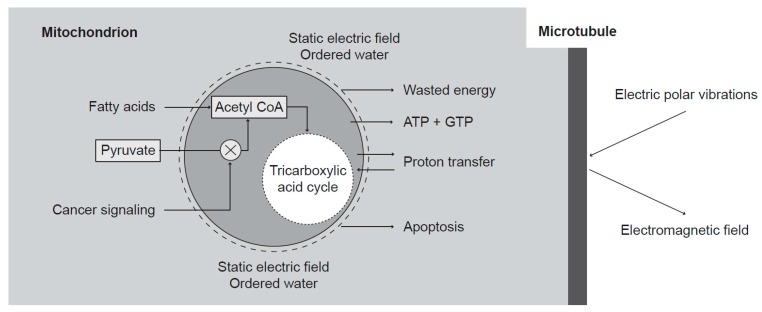
Transformation of the biochemical disturbance along the cell transformation pathway into the biophysical pathological states. Mitochondria transport protons into the intermembrane space, and their diffusion into cytosol leads to generation of a strong static electric field and water ordering. The strong static electric field shifts also vibrations in microtubules into a highly nonlinear region. The decrease of oxidative metabolism results in changes of the intensity of the static electric field and changes of water ordering. The water ordering depends on pH factor and also on the static electric field (Figure 1). Energy is supplied to microtubules by hydrolysis of GTP upon β-tubulin polymerization, through motion of motor proteins, and by nonlinear transfer from the higher frequency oscillations.

The generated electric oscillating field was measured through an attraction of small dielectric particles with high permittivity and linear dimensions of about 1 μm by yeast cells. The dielectrophoretic attraction showed the highest power in the M phase [39]. The M phase increased activity of yeast cells in the frequency region of 8–9 MHz was confirmed by Pokorný *et al.* [40]. Microtubules were assumed to represent the oscillating structure of EMG activity [31]. Advanced computer simulation of the generated EMG field describes its characteristic properties [41]. Sahu *et al.* [42,43] measured the resonant frequencies of isolated microtubules in the frequency range of about 0.1–0.4 MHz, 10–30 MHz, 100–200 MHz, 1–20 GHz, at approximately 20 THz (the wavenumber about 700 cm^−1^) (Table 1), and the UV absorption-emission spectrum at about 276 nm. Disturbances of microtubule oscillations are assumed to form a link along the transformation pathway to cancer (Figure 3).

**Table 1:** Spectral resonant components of microtubule oscillations. (*f*—frequency, *λ*—wavelength, *ν—*wavenumber). After [42,43]. Absorption-emission spectra at 276 nm are not included. (a) In the classical frequency bands below 20 GHz; (b) In the far-infrared region.

(a) In the classical frequency band below 20 GHz											
Quantity		Unit		Measured values							
*f*		kHz		120		240		320			
*λ*		m		2500		2143		940			
*f*		MHz		12		20		22		30	
*λ*		m		25		15		13.6		10	
*f*		MHz		101		113		185		204	
*λ*		m		2.96		2.65		1.6		1.5	
*f*		GHz		3		7		13		18	
*λ*		cm		10		4.3		2.3		1.7	
(b) In the far-infrared region											
	Quant.		Unit	Measured values							
Large amp.	*ν*		cm^−1^	526				682			
	*f*		THz	15.8				20.5			
Small amp.	*ν*		cm^−1^	307	335		850	948	1041	1427	1497
	*f*		THz	9.2	10.0		25.5	28.4	31.3	42.8	44.9

The power supply to the electric polar vibrations in a cell is assumed to be of about 0.1 pW (10^−13^ W). Assuming that the number of microtubules in a cell is 400, then the power supply to a single microtubule is of the order of magnitude of 0.1 fW (10^−16^ W). For a quality factor of about 80, the power of electric polar vibrations in one microtubule is about 10 times higher (*i.e.*, 1 fW). The EMG component of oscillations is therefore smaller than 1 fW.

## 5. Cancer Initiation

Cancer may be initiated by oncogene mutations, an event of thousands of random somatic mutations, which can be identified in a single cancer sample. Most of the mutations are only “passengers” which do not bear significant information about cancer [44]. Storing data in DNA should be feasible to preserve information gained during evolution. DNA may be considered as a memory system of permanent localized storage capability, with a limited access controlled by a special cellular mechanism aimed at preventing random events. Genome mutations imply disturbances of the storage-controlling mechanism, and may lead to various diseases, including cancer [44,45,46,47,48,49].

Mechanism controlling the storing and reading exerts a crucial cellular function. Cell-mediated immunity (CMI) is considered to correspond to the adherence of T-lymphocytes to solid state surfaces. The CMI response of T-lymphocytes was measured in healthy women and in patients with cervical precancerous lesions and cervical cancer, by the leukocyte adherence inhibition (LAI) assay. To this aim, both the cancer antigen prepared from cervical carcinoma tissue and the non-specific antigen prepared from lactate dehydrogenase (LDH) virus-infected mice blood were used [50,51]. The CMI response was elicited in all investigated cancers and the majority of T-lymphocytes taken from cancer patients did not adhere, whereas those from healthy women did. These results suggest that the mechanism of genome protection is disturbed by infection of the LDH virus, which was wrongly assumed to be silent and not harmful to the host. Chronic parasitic energy consumption in the cell is provided by its single stranded RNA. Basic information on LDH virus is provided by experimental studies in mice [52,53]. Infection with LDH virus results in increased level of the LHD enzyme (now classified as NAD 1.1.1.27 Oxidoreductase) in plasma and a production of lactate from pyruvate. Parasitic energy consumption by the LDH virus RNA may affect the cellular oxidative metabolism and result in decreased power and coherence of polar vibrations. It may be concluded that somatic mutations are a consequence of LDH virus infection or of an infectious agent eliciting similar CMI response.

Upon occurrence of oncogene mutations, oncogene–induced senescence (OIS) is abrogated, which in turn leads to activation of the pyruvate dehydrogenase kinases PDK1–4 and inhibition of the pyruvate dehydrogenase phosphatases PDP1–2. Kolobova *et al.* [27] reported that the enzymatic activity of mammalian pyruvate dehydrogenase is regulated through three phosphorylation sites—serine residues Ser-264, Ser-271, and Ser-203.

Due to technological advances, a large amount of different mutations can now be detected in a single cancer sample, which makes it possible to decode mutational signatures of particular cancer types. Alexandrov *et al.* [44] catalogued signatures of somatic mutations causing human cancers, but the biological processes leading to mutation occurrence are poorly understood. The role of mutated oncogenes in various cancers is a research topic of wide interest [45,46,47,49]. Oncogene mutations may result in mitochondrial dysfunction [54].

## 6. Interaction between Cells

The enslavement of a cell within a tissue depends on its EMG activity. In a tissue, cells are mutually controlled by intercellular interactions that can be described by a physical EMG mechanism [25,55,56]. The main factors for effective interactions are the frequency spectrum, its space pattern, and the power of the EMG field generated by the interacting cells. If the frequency spectrum and the space pattern of the EMG field of cancer cells and tissue are different, cells may perform individual activities and escape from interactions. The space pattern depends on the geometrical arrangement of the sources (*i.e.*, microtubules) and their surrounding parts, *i.e.*, other cytoskeleton structures, and on the ordering of water molecules. Some differences between the properties of normal and tumor cells are mentioned. The keratin network around the nucleus undergoes shrinking after exposure to the bioactive lipid sphingosylphosphorylcholine (SPC) [57] and consequently affects microtubule oscillations. Interaction forces between normal cells and between normal and cancer cells may significantly differ [6]. Local invasion and metastasis may depend on altered force effects and disturbances of the intercellular matrix. This process is similar to the epithelial-mesenchymal transition.

Two types of cancer process have been examined by measuring the mitochondrial inner membrane potential. Mitochondria in tumor cells with a glycolytic phenotype and inhibited pyruvate transfer display an apparent hyperpolarization of the membrane. This phenotype, corresponding to the Warburg effect, was found in the vast majority of adenocarcinomas, carcinomas, and melanomas, but not in certain cancer types, suggesting a modified glycolytic phenotype. This phenotype, called the reverse Warburg effect [58], was observed, for instance, in lymphomas, breast and prostatic cancers, and may exist in many different epithelial tumors. Mitochondria in fibroblasts associated with cancer cells are dysfunctional and energy-rich metabolites such as lactate, pyruvate, glutamine, keton beta-hydroxybutyrate (BHB) are supplied from fibroblasts to cancer cells with fully functional mitochondria. The main characteristics of the reverse Warburg effect in cancers have been previously addressed in detail [59].

Disturbances of the coherent energy states far from the thermodynamic equilibrium are considered the essential process of cancer [60,61]. The mitochondrial dysfunction in cancer cells or in cancer cell-associated fibroblasts in stroma affects cell transformation and tumor development. The EMG field generated by microtubules is a function of the mitochondrial activity [25,62,63,64]. In normal cells, mitochondria supply energy and provide low damping of oscillations due to the ordered water molecules around them. The oscillation frequencies and coherence depend on the power and potential valley of the oscillator and its non-linear characteristics. If the force coefficient in the potential valley increases with the decreasing oscillation power, the frequency increases and coherence decreases. If the potential valley has a flat region at a high power, the frequency decreases with the increasing power and small power deviations may cause large disturbances of coherence. The absorption resonant frequencies of some tumors were measured around 465 MHz [65], which should correspond to the shifted spectral lines of normal cells.

**Figure 4:**
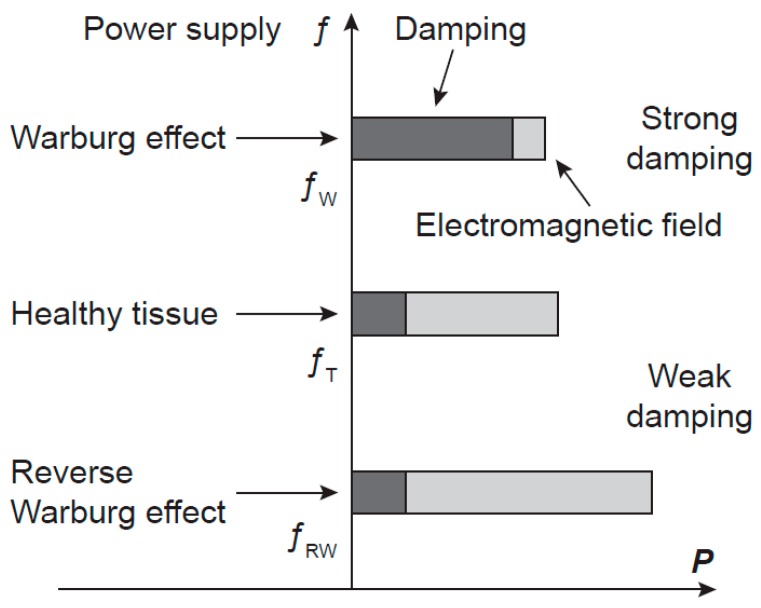
A hypothesis of the power-frequency relation of oscillations in cancer cells. A schematic picture of the frequency *f* and of the power *P* of the electromagnetic field generated by microtubules in normal and cancer cells. Cancer cells with the Warburg and the reverse Warburg effects generate electromagnetic fields with lower and higher power than cells in a healthy tissue, respectively, causing frequency shifts in opposite directions. The generated power is determined by energy supply and damping. *f*_T_, *f*_W_, and *f*_RW_ are frequencies of microtubule oscillations of normal cell and cancer cells with the Warburg and the reverse Warburg effect, respectively.

In the case of cancerous cells characterised by the Warburg effect, the power of microtubule oscillations and of the generated EMG field is down-regulated and their parameters are altered. Coherence of the EMG field may be disturbed, the frequency spectrum changed and shifted from the tissue frequency region (in correspondence with the decreased power). In the case of the reverse Warburg effect, the enhanced mitochondrial activity and supply of energy-rich metabolites to cancer cells provide increased power of oscillations, changed frequency spectrum and its shift outside the tissue region too (in correspondence with the increased power). Consequently, in both types of Warburg effects, cancer cells may escape from tissue control and regulations and begin their activity independently of the tissue. Hypothetical scheme of power, damping, and frequency of cancers with the Warburg and the reverse Warburg effect are depicted in Figure 4.

Notably, disturbances of the coherent EMG field in cancer cells may explain the asbestos’ carcinogenicity. Asbestos forms optic fibers in the UV band and conducting wires after adsorption of conducting molecules containing, for instance, iron atoms [66,67]. EMG field in the cell is short-circuited by the asbestos fibres.

## 7. Biophysical Insight into Cancer

**Figure 5:**
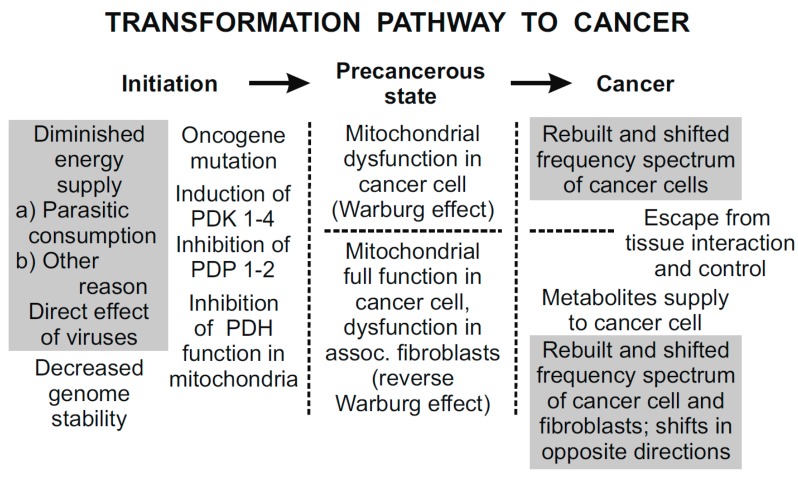
The three principal periods of the transformation pathway to cancer [60]. Hypothetical links supported by experimental results are in grey rectangles. The remaining links are experimentally proven. In the first period, initiation, diminished energy supply (e.g., by parasitic energy consumption) results in decreased genome stability. Somatic mutations may include oncogene mutations that may result in impaired mitochondrial signaling. In the second period, precancerous state, mitochondrial dysfunction results in biophysical effects; coherent EMG activity of inflicted cells is down-regulated. In the final period, cancer, tumor cells with down- and up-regulated EMG activity are independent of the tissue control.

Disturbances of oxidative metabolism result in pathological states. Pathological agents may directly affect the mitochondrial oxidative activity or the microtubule oscillating systems, or indirectly, as a consequence of biochemical and genetic defects. Carcinogenesis comprises a wide spectrum of mechanisms, from genetic to biochemical, to biophysical [55,56,64], and any partial knowledge may be misleading in understanding the whole process. Cancer is one of the diseases caused by a defective energy system. The transformation pathway to cancer is shown in Figure 5 [60].

In the first period, the initiation, the reduced energy supply caused by RNA of LDH virus or similar agents is assumed to result in decreased genome stability and DNA mutations. However, the mechanisms leading to these mutations and their differences in the Warburg effect and in the reverse Warburg effect cancers remain unclear. One of the essential conditions for creating the reverse Warburg effect is the loss of stromal Caveolin-1 [58,68,69,70,71,72], which is connected to the induction of oxidative stress, autophagy/mitophagy [68,72,73], and mitochondrial dysfunction in the associated fibroblasts [58].

In the second period, the precancerous state, two cancer phenotypes are mainly established. The Warburg and the reverse Warburg effect cancer cells may have either dysfunctional or fully functional mitochondria, respectively. In the latter case, tumor growth and metastasis are supported by the supply of energy-rich metabolites from the associated fibroblasts [58,68,71,74,75] with dysfunctional mitochondria. In this case, transformation is a tissue-related process.

In the last period, cancer, transformed cells start performing their activities independently of the tissue. The frequency of EMG activity is shifted to the direction corresponding to generated power. In case of the reverse Warburg effect, the frequency shifts of tumor cells and associated fibroblasts are in opposite directions, which enables local invasion and metastasis in the early phase of their development. The high energy level of cancer cells explains their aggressiveness.

## 8. Discussion

Coherent electrodynamic state excited and maintained by energy supply is a nature of life. In eukaryotic cells, the electrodynamic state is formed by coherent electric polar vibrations of microtubules. Disturbances of the coherent state by insufficient energy supply, dysfunction of mitochondria, defects of microtubules and water ordering result in pathological conditions. Experimental investigations suggest inadequacy of energy dependent processes detected by CMI response to LDH virus antigen in patients with cancer, women displaying recurrent spontaneous abortions in early pregnancy from unknown reasons and women who have given birth to stillborn babies with organ malformations, patients with acute myocardial infarction, and schizophrenia [76,77]. Microtubule vibrations were proposed to be dominant in the brain function [60], whereby consciousness is based on the coherent electrodynamic state and evaluation of its difference between a living system and its surroundings performed by the orchestrated objective reduction. Disturbances of the coherent electrodynamic state result in various neurological disorders whose origin has been questionable including Schizophrenia. Dependence of the brain function on quantum electrodynamic processes, self-collapse, and orchestrated objective reduction of the quantum states to a simple classical state are analyzed in [78,79,80].

Cancer is also a pathology of a disturbed coherent electrodynamic state. Liability of the genome system to undergo a genome somatic mutation and aggressiveness of carcinogens determine the possibility of cancer initiation. The role of LDH virus activity in cancer initiation is supported by CMI measurements [50,51]. Rubella virus is also recognized as being a teratogenic virus. The genome mutation by viruses is a result of infection. Oncogens are mutated as a by-product of mass non-localized genome mutation [44] which signifies disturbance of the mechanism controlling storage. Mechanism restricting manipulation of the DNA memory is not yet understood.

To date, cancer initiation remains poorly understood, along with the defects of cell interactions within a tissue causing tumors of the reverse Warburg effect. Similarly, the knowledge of normal Warburg effect cancers is unclear, and in particular the cause of increased probability of somatic mutation occurrence. It is claimed that most of the variations in cancer are due to “bad luck”, *i.e.*, to random events [81]. Experimental evidence indicates a role in causing genome mutations for LDH virus infections or infectious agents eliciting similar CMI responses [50,51].

The essential property of living systems is a coherent state far from thermodynamic equilibrium. Physiological levels of the coherent state depend on oxidative metabolism. Mitochondrial activity may be reduced upon low pyruvate supply, induced, for example, by parasitic consumption, or by blocking PDH enzymes. RNA of the LDH virus parasitically consumes energy in the cell. The effects of RNA on genome mutations may be mediated by mitochondria and/or caused by an unknown mechanism controlling chemical reactions.

The mechanism of mitochondrial dysfunction in cancer development deserves elucidation. Electric polarization of the mitochondrial inner membrane depends mostly on the production of reactive oxygen species, proton transfer across the inner membrane, and the distribution of ions in the cell. Inhibition of pyruvate transfer into mitochondrial matrix causes a step decrease of proton transfer and potential barrier across the inner membrane. Arrangement of the ordered water is shifted from the point of a normal pH value smaller than the value in the point of zero thickness to a pH value higher than that in the point of zero thickness (Figure 1), *i.e.*, the exclusion of positively and negatively charged entities is exchanged. The shift is confirmed by measurement of mitochondrial inner membrane potential by uptake and retention of positively charged fluorescent dyes and their high accumulation around dysfunctional mitochondria, which is overviewed and analysed in [59]. Damping of the electrodynamic oscillations might be caused by the mobile negatively charged entities (electrons) at the upper boundary of the exclusion layer. Healthy cells generate energy from oxidative breakdown of several substrates including pyruvate and glutamine [82]. In transformed mammalian cells, glutamine-driven oxidative phosphorylation is the major source of high-energy electrons [83]. However, in transformed cells glutamine is often redirected into anabolic pathways [84]. On the other hand, glutamine reduces the oxidative stress, stops the process of cancer cachexia, and nourishes the immunological system. It supports the organism to fight against the cancer rather than the cancer cells [85]. In the Warburg effect cancer cells, a step increase of glutamine production of ATP and GTP to overcome pyruvate inhibition might restore mitochondrial function. In the reverse Warburg effect cancer cells, the transport of energy rich metabolites including glutamine from associated fibroblasts should be inhibited.

The short overview of the mitochondrial dysfunction along with the transformation pathway to cancer included in this article enables assessment of novel methods for cancer eradication. In general, therapeutic strategies targeting fast proliferating cells display a lack of specificity, resulting in toxic effects on normal cells. Bonnet *et al.* [86] tested the effects of dichloroacetate *in vitro*, demonstrating the feasibility to restore mitochondrial function, and therefore provided evidence that it is a promising selective anti-tumor agent. Moreover, Tennant *et al.* [87] suggested treatment by targeting tumor specific enzyme isoforms which changes their activity or concentration of the substrate. Targeting oxidative metabolism may be considered as a promising therapeutic strategy for the treatment of cancer. The pyruvate pathway is restored after dephosphorylation of three serine residues of the PDH enzyme. However, this method by itself only temporarily removes a link along the cell transformation pathway (eventually leading to cancer). Steady induction of PDKs and inhibition of PDPs after mutations of oncogenes may inactivate PDH again and reproduce mitochondrial dysfunction. Therefore, if OIS is abrogated, the cancer phenotype may be restored. However, even in this case the repeated temporary restoration of PDH function in mitochondria might very likely lead to positive results by triggering apoptosis of severely damaged cells. Establishment of normal OIS could enable steady restoration of a normal state and apoptotic function of the cell. Together, these data support the reversal of mitochondrial dysfunction as a possible strategy for cancer treatment.

Killing strategies may be effective if a selective targeting on cancer cells is disclosed. Differentiation of normal and tumor cells might be based on detection of the level of EMG coherence and/or frequency spectrum of the cell. In comparison with normal cells, the spectrum of the EMG field of cancer cells is rebuilt and the frequency changed. The frequency spectra may depend on development of cancer and its stages. Vedruccio and Meessen [65] measured the frequency spectra of some cancer tissues at about 465 MHz. Synthesis of molecules oscillating at least at one frequency of the cancer cell spectrum can mediate attractive interaction with the cancer cells [88]. The synthesized molecule may transport the anti-tumour drug and selectively target the cancer cell. Selectivity of treatment may also be based on the difference of the oxidation potential between healthy and cancer cells.

Application of electromagnetic fields for cancer treatment encounters serious obstacles regardless of selectivity in the case of resonance. A small signal electromagnetic field need not be effectively absorbed due to symmetry of the cellular generating system preventing loss of power and information transfer into the cell surroundings in the case of a separated cell or ordering corresponding to interaction with the surrounding cells in the case of a cell in the tissue. Nevertheless, absorption of electromagnetic signals can be used for cancer diagnostics [65]. A weak sinusoidal magnetic field affects the cell mediated immunity [89]. It cannot be excluded that small signal electromagnetic resonances can trigger some important processes in a living cell.

Killing strategy by electromagnetic heating can be selective in the case of resonance. However, in nonlinear systems the resonant frequency depends on energy stored in the oscillating systems. Therefore, the frequency of the emitted signal for treatment should be continually adjusted to the highest absorption of the near electromagnetic field in the cancer cells similarly as in cancer diagnostics [55,65]. The overheating of the surrounding tissue by heat conduction and blood transfer should be prevented.

## 9. Conclusions

Continuous energy supply to a biological system is a condition *sine qua non* for existence of life. Biological systems employ energy for building morphological structures, excitation of energy-rich processes, electrostatic, vibrational, and EMG fields. In this way, a state far from thermodynamic equilibrium is achieved. Ordered water molecules in living cells provide low damping of microtubule electric polar vibrations. In cancer cells or in fibroblasts associated with cancer cells characterized with the Warburg or the reverse Warburg effect, respectively, mitochondrial dysfunction results in disturbed water ordering and damped vibrations. As cell interactions depend on the generated EMG field, an altered frequency spectrum of the EMG field of cancer cells or fibroblasts, compared to that of normal cells, is a condition for cancer local invasion and metastasis.

Cancer is a pathological impairment of coherent energy states. In both basic phenotypes of cancer, the Warburg and reverse Warburg effect, induction (inhibition) of pyruvate dehydrogenase kinases (phosphatases) contributes to confer a unique metabolic profile, characterized by increased glycolysis and suppression of mitochondrial pyruvate oxidation, which results in local invasion, metastasis, and reduced apoptosis. Restoration and sustaining of normal mitochondrial functions in cancer cells or associated fibroblasts may be a promising anti-cancer therapeutic strategy.
